# Onset of Guillain-Barre Syndrome and Transverse Myelitis Following COVID-19 Vaccination

**DOI:** 10.7759/cureus.41009

**Published:** 2023-06-26

**Authors:** Kenny Do, Eric Kawana, Jenifer Do, Luis Diaz

**Affiliations:** 1 Department of Internal Medicine, Kirk Kerkorian School of Medicine at University of Nevada, Las Vegas (UNLV), Las Vegas, USA; 2 School of Life Sciences, University of Nevada, Las Vegas (UNLV), Las Vegas, USA; 3 Department of Neurology, MountainView Hospital, Las Vegas, USA

**Keywords:** bilateral lower limb paralysis, covid-19 vaccine, vaccine-associated transverse myelitis, guillain-barre syndrome (gbs), covid-19

## Abstract

Guillain-Barre syndrome (GBS) and transverse myelitis (TM) are both neuro-inflammatory disorders that are attributed to dysfunctions of the peripheral nervous system and spinal cord, respectively. The two conditions involve immune-mediated destruction and inflammation of the nervous system and may present clinically as a weakness in the muscles, loss of normal sensations, and even paralysis of the body or extremities. Although the incidence of GBS and TM is quite rare, there have been reports of the two diseases developing in patients, either independently or concurrently, following COVID-19 vaccinations. In this case report, we present a patient (male) who lost functions and sensations in his lower extremities 60 days after he received the COVID-19 booster vaccine. The patient’s blood work was unremarkable. Magnetic resonance imaging (MRI) of his thoracic spine and an electromyography study revealed evidence of nerve demyelination, which supports the diagnosis of GBS/TM overlap syndrome. He was ultimately treated with intravenous immunoglobulins (IVIGs) and gained back functions in his lower extremities.

## Introduction

Guillain-Barre syndrome (GBS) is an autoimmune condition that involves muscle numbness and even paralysis due to immune-mediated degeneration of the peripheral nerves [1]. Although it is the leading cause of acute flaccid paralysis, GBS is quite rare, with an incidence ranging from 0.04% to 0.4% [2]. The exact mechanism of GBS is relatively unknown; however, there are many proposed theories regarding the pathogenesis of the disease, which include autoimmune destruction, molecular mimicry, and peripheral nerve inflammation [3]. All of these proposed mechanisms have a common physiological response involving antibodies attaching to and destroying Schwann cells [4].

Similarly, transverse myelitis (TM) is a neurological condition that presents with an immune-mediated attack and inflammation of the spinal cord [5]. This disorder may involve demyelination and inflammation of oligodendrocytes, myelin, axons, and even neurons because the white and gray matter of the nervous system is affected [5]. Patients with this disease may experience gradual muscle sensory loss, motor weakness, and even paralysis [6]. TM is a rare condition, with one to eight people per 1 million individuals developing the disease annually [5].

GBS and TM may both present with similar symptoms and may both coexist in patients following COVID-19 infection and vaccination, known as GBS/TM overlap syndrome [4,6,7]. Vaccinations against SARS-CoV-2 are mostly based on the messenger ribonucleic acid (mRNA) and viral vector platforms, where common symptoms following vaccinations include fevers, chills, nausea, headaches, and more [8,9]. Although there have been reports of isolated cases of GBS and TM following COVID-19 vaccinations, this case report presents a rare phenomenon where a patient presented with both syndromes after receiving a COVID-19 booster vaccine.

## Case presentation

The patient was a 59-year-old male who consulted with a neurologist after developing paraplegia. He shared that he was healthy and did not experience any major symptoms before March 2022. He reported that he suddenly developed lower back pain and soon after experienced numbness and tingling of the bilateral lower extremities, which later ascended to his hips. The patient subsequently experienced urinary retention, constipation, and lower extremity weakness. After two weeks, the patient was unable to walk and lost functions in his legs. Sixty days before the onset of his symptoms, the patient had received a COVID-19 booster vaccine.

After the progressive onset of his symptoms, the patient was admitted to the hospital and was diagnosed with TM. MRI of his cervical spine demonstrated signs of osteoarthritis with the spinal cord intact. Mild stenosis of the spinal canal was also found, although there was no compression to the spinal cord. The MRI of the brain was unremarkable, while the MRI of the patient's thoracic spine revealed patchy hyperintensities consistent with demyelination of the thoracic spinal cord. Blood work performed at the hospital was also unremarkable and normal, with negative results for infections such as Lyme disease or syphilis. At the hospital, the patient was given plasmapheresis and steroids intravenously, although his condition did not respond to the therapy.

**Figure 1:**
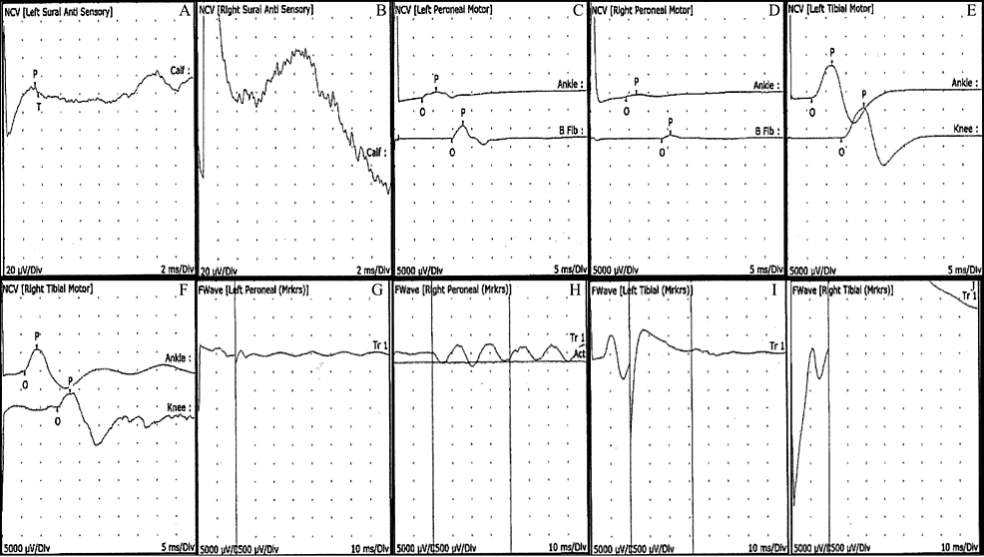
Waveforms of the sural, peroneal, and tibial nerves: (A) NCV (left sural antisensory); (B) NCV (right sural antisensory); (C) NCV (left peroneal motor); (D) NCV (right peroneal motor); (E) NCV (left tibial motor); (F) NCV (right tibial motor); (G) F-wave (left peroneal [markers]); (H) F-wave (right peroneal [markers]); (I) F-wave (left tibial [markers]); (J) F-wave (right tibial [markers]). NCV, nerve conduction velocity

Because the treatments at the hospital did not work, the patient then consulted with a neurologist in an outpatient setting. Upon examination with the neurologist, the patient’s cranial nerves II-XII were intact and motor functions to his face and upper extremities were normal. He had some degree of impairments to his bowel and urinary functions. Motor functions to his legs were absent although he was able to feel some vibrations. Nerve conduction and electromyography studies were performed in his lower extremities, which revealed demyelination and dysfunctions in the engagement of distal muscles below the knee (Tables 1-2). The F-wave in the right tibial motor nerve was absent, while the F-wave in the left tibial motor nerve was normal (Table 3). The F-waves in the peroneal nerves were abnormal as well, with the left one being absent and the right one being prolonged (Table 3). All of these findings, along with other testing and waveforms, suggested the coexistence of GBS with TM (Tables 1-4 and Figure 1). EMG testing of the thigh muscles demonstrated normal recruitment with no signs of denervation. 

**Table 1:** Motor summary table showing abnormal nerve conduction velocities in the lower extremities. ^*^Abnormal findings.

Stimulation site	Nerve/Muscle	Onset (ms)	Normal onset (ms)	O-P amplitude (mV)	Normal O-P amplitude	Site 1	Site 2	Delta-0 (ms)	Distance (cm)	Velocity (m/s)	Normal velocity (m/s)
Ankle	Left peroneal motor (extensor digitorum brevis)	7.1*	<5.5	1.6*	>2.5	Fibular head	Ankle	7.7	31.0	40	﻿>38
Fibular head	Left peroneal motor (extensor digitorum brevis)	14.8	<5.5	3.4	>2.5						
Ankle	Right peroneal motor (extensor digitorum brevis)	9.2*	<5.5	0.5*	>2.5	Fibular head	Ankle	9.2	32.0	35*	﻿>38
​​​​​​​Fibular Head	Right peroneal motor (extensor digitorum brevis)	18.4	<5.5	0.9*	>2.5						
Ankle	Left tibial motor (abductor hallucis brevis)	6.3*	<6.0	8.7	﻿>3.0	Knee	Ankle	7.5	32.0	43	>40.8
Knee	Left tibial motor (abductor hallucis brevis)	13.8	<6.0	8.2	﻿>3.0						
Ankle	Right tibial motor (abductor hallucis brevis)	5.9	<6.0	5.8	﻿>3.0	Knee	Ankle	8.4	32.0	38*	>40.8
Knee	Right tibial motor (abductor hallucis brevis)	14.3	<6.0	3.6	﻿>3.0						

**Table 2:** EMG table showing reduced recruitment in the lower extremities *Abnormal findings. EMG, electromyography

Side	Muscle	Nerve	Root	Insertional activity	Fibrillations	Positive short waves	Amplitude	Duration	Polyphasic potentials	Recruitment	Interference pattern
Right	Peroneus longus	Superficial peroneal nerve	L5-S1	Normal	Normal	Normal	Normal	Normal	0	Reduced*	25%*
Right	Anterior tibialis	Deep peroneal nerve	L4-5	Normal	Normal	Normal	Normal	Normal	0	Reduced*	25%*
Right	Posterior tibialis	Tibial	L5, S1	Normal	Normal	Normal	Normal	Normal	0	Reduced*	25%*
Right	Vastus lateralis	Femoral	L2-4	Normal	Normal	Normal	Normal	Normal	0	Reduced*	50%*
Right	Vastus medialis	Femoral	L2-4	Normal	Normal	Normal	Normal	Normal	0	Reduced*	50%*
Right	Lateral gastrocnemius	Tibial	S1-2	Normal	Normal	Normal	Normal	Normal	0	Reduced*	25%*
Right	Medial gastrocnemius	Tibial	S1-2	Normal	Normal	Normal	Normal	Normal	0	Reduced*	25%*
Right	Biceps femoris (long head)	Sciatic	L5-S2	Normal	Normal	Normal	Normal	Normal	0	Reduced*	25%*
Left	Extensor digitorum brevis	Deep peroneal nerve	L5, S1	Normal	Normal	Normal	Normal	Normal	0	Reduced*	25%*
Left	Peroneus longus	Superficial peroneal nerve	L5-S1	Normal	Normal	Normal	Normal	Normal	0	Reduced*	25%*
Left	Anterior tibialis	Deep peroneal nerve	L4-5	Normal	Normal	Normal	Normal	Normal	0	Reduced*	25%*
Left	Vastus lateralis	Femoral	L2-4	Normal	Normal	Normal	Normal	Normal	0	Reduced*	75%*
Left	Vastus medialis	Femoral	L2-4	Normal	Normal	Normal	Normal	Normal	0	Reduced*	75%*
Left	Lateral gastrocnemius	Tibial	S1-2	Normal	Normal	Normal	Normal	Normal	0	Reduced*	25%*
Left	Medial gastrocnemius	Tibial	S1-2	Normal	Normal	Normal	Normal	Normal	0	Reduced*	25%*

**Table 3:** F-wave studies showing prolonged or absent latencies. ^*^Abnormal findings. NR, no response

Nerve/Muscle	NR	F-wave latency (ms)	Latency normal (ms)
Left peroneal (markers) (extensor digitorum brevis)	NR*		<60
Right peroneal (markers) (extensor digitorum brevis)		60.27*	<60
Left tibial (markers) (abductor hallucis)		51.63	<61
Right tibial (markers) (abductor hallucis)	NR*		<61

**Table 4:** Left/Right comparison of the peroneal and tibial motor nerves. ^*^Abnormal findings.

Stimulation site	Nerve/Muscle	Left latency (ms)	Right latency (ms)	Left-right latency (ms)	Left amplitude (mV)	Right amplitude (mV)	Left-right amplitude (%)	Site 1	Site 2	Left velocity (m/s)	Right velocity (m/s)	Left-right velocity (m/s)
Ankle	Peroneal motor (extensor digitorum brevis)	7.1*	*9.2	2.1*	1.6*	0.5*	68.8*	Fibular head	Ankle	40.0	35*	5
Fibular head	Peroneal motor (extensor digitorum brevis)	14.8	18.4	3.6	3.4	0.9*	73.5					
Ankle	Tibial motor (abductor hallucis)	6.3*	5.9	0.4	8.7	5.8	33.3	Knee	Ankle	43.0	38*	5
Knee	Tibial motor (abductor hallucis)	13.8	14.3	0.5	8.2	3.6	56.1					

For his treatment plan, the patient was given intravenous immunoglobulin (IVIG) 400 mg/kg for five days as a loading dose followed by 1 g/kg every three weeks. Since receiving his initial loading dose and subsequent IVIG treatments four weeks after consulting with the neurologist, the patient demonstrated significant improvements, with him being able to stand and ambulate with a walker. Just over six months after his initial treatment, the patient gained normal strength and functions in his lower extremities, aside from experiencing mild issues with his balance and vibrational perception. The patient is expected to improve, and he will continue the same treatment regimen.

## Discussion

Patient development of GBS or TM following vaccinations is not new and not exclusive to only COVID-19 vaccines. A systematic review that examined literature up to 2020 shared that GBS was reported in 58 different studies following influenza, hepatitis, polio, rubella, measles-rubella, measles-mumps-rubella (MMR), and other types of vaccines [10]. Similarly, another systematic review demonstrated 37 studies in which TM resulted following MMR, hepatitis B, diphtheria-tetanus-pertussis (DTaP), and other types of vaccines [6,11]. It is hypothesized that the development of neuro-inflammatory syndromes like GBS or TM following COVID-19 or other vaccinations is attributed to molecular mimicry [6]. In this pathway, foreign antigens in the vaccines present similarly to host cells and tissue, where this leads to an immune and inflammatory response that leads to the destruction of both foreign and host structures [6].

There are four main subtypes of GBS, and the most common one is the acute inflammatory demyelinating polyradiculoneuropathy variant [3]. This subtype involves the destruction of myelin sheaths in nerve cells, leading to the clinical presentation of bilateral muscle weakness, diminished reflexes, and acute flaccid paralysis [3,9]. Typically, these symptoms start in the lower extremities and gradually ascend to other muscles. Patients developing GBS may present with impairments to their cranial nerves and lungs as well [12]. An investigation from the CDC revealed that the incidence of GBS for adults in the United States within 21 days of vaccination was 0.29 per 1,000,000 for the Pfizer vaccine, 0.35 per 1,000,000 for the Moderna vaccine, and 3.29 per 1,000,000 for the Janssen vaccine [13].

The development of TM usually occurs acutely or subacutely, and the causes of the syndrome may occur during or after an infection, although most cases of TM are idiopathic [5,9]. Similar to GBS, the pathophysiology of TM involves nerve demyelination and inflammatory infiltration of the spinal cord, leading to neurologic impairments [5]. Patients may experience different symptoms depending on where the nerves are destroyed in certain spinal cord regions. Dysfunction of the nerves in the cervical region may lead to paralysis of all four limbs and respiratory impairment, whereas destruction of nerves in the lumbosacral region may cause sensory deficits and motor loss in the legs [5]. However, most cases of TM occur in the thoracic region of the spinal cord. Recent studies have suggested that the incidence of TM related to COVID-19 infection is 0.5 per million cases [14].

It is believed that GBS is the second most common and TM is the fourth most common critical neurological side effect following COVID-19 vaccinations [9]. However, the existence of the two syndromes occurring together, known as GBS/TM overlap syndrome, is presumed to be rare. Most cases of this syndrome are related to recent infections, such as from the *Zika* virus or *Mycoplasma* bacteria [7]. Little is known regarding the most effective treatments to address the coexistence of GBS and TM, although current literature suggests that the use of IVIG is effective at addressing the overlap syndrome [15].

## Conclusions

COVID-19 vaccines have been effective in containing the spread of SARS-CoV-2 during the pandemic and in preventing severe medical complications following infection. Like many other vaccines, adverse side effects may develop in patients following vaccinations, such as neurological dysfunctions. However, it is important to note that the advantages of receiving COVID-19 vaccines outweigh the potential side effects, which are usually benign. Although the incidences of GBS, TM, or GBS/TM overlap syndrome are rare, physicians should still be aware of these complications and treat them appropriately when they present in patients following COVID-19 vaccinations.
